# Clinical Study of Intestinal Pacemaker Cells in Neonates With Small Bowel Atresia

**DOI:** 10.7759/cureus.97522

**Published:** 2025-11-22

**Authors:** Anusiri Inugala, Satyanarayana Ravula

**Affiliations:** 1 Pediatric Surgery, Institute of Child Health Niloufer Hospital, Hyderabad, IND; 2 Pediatric Surgery, Ankura Hospital for Women and Children, Hyderabad, IND

**Keywords:** immunohistochemistry, interstitial cells of cajal, neonatal intestinal obstruction, postoperative outcome, small bowel atresia

## Abstract

Background: Small bowel atresia is a leading cause of neonatal intestinal obstruction and is frequently associated with postoperative gastrointestinal dysmotility. While factors like enteric nervous system alterations have been studied, the role of interstitial cells of Cajal (ICCs), the gut's pacemaker cells, remains under-investigated.

Objective: This study aimed to assess the density and distribution of ICCs in the atretic, proximal, and distal bowel segments in neonates with small bowel atresia and correlate their presence with postoperative outcomes.

Methods: A prospective study was conducted over two years at Institute of Child Health Niloufer Hospital, Hyderabad, including 44 neonates diagnosed with various types of small bowel atresia. Resected bowel segments were analyzed histologically and immunohistochemically using anti-CD117 (c-Kit) antibodies to identify ICCs. Clinical parameters such as age, type and location of atresia, postoperative bowel function, and survival were documented and statistically correlated with ICC distribution.

Results: Among the 44 neonates (52.27% female), jejunal and ileal atresias were most common. The majority presented within the first five days of life. CD117 staining revealed sparse or absent ICCs in the atretic and proximal segments in neonates with delayed bowel function and poorer outcomes. In contrast, higher ICC expression was significantly associated with earlier stool passage and improved survival (p < 0.05).

Conclusion: A reduction in ICCs is a notable pathological feature of small bowel atresia. Their density in resected bowel segments significantly influences postoperative bowel motility and survival. ICC density assessment may serve as a prognostic indicator and guide surgical planning in affected neonates.

## Introduction

One of the most common surgical emergencies with which a neonate presents to the surgical NICU is neonatal small bowel obstruction. Intestinal atresia accounts for approximately one-third of all cases of neonatal intestinal obstruction [1].

Intestinal atresia is responsible for nearly one-third of all neonatal cases of intestinal blockage [2]. Newborns with obstruction of the small intestine can exhibit a range of symptoms, such as greenish (bilious) vomiting, abdominal bloating, inability to pass meconium, and sometimes jaundice. Diagnosis typically involves a thorough clinical evaluation, plain abdominal radiographs, and contrast enema studies. The underlying cause, clinical features, diagnostic approach, surgical treatment, postoperative management, and prognosis can differ significantly depending on the site of the obstruction.

The causes, clinical presentation, diagnosis, operative management, postoperative care, and outcome may vary considerably according to the location of the obstruction.

The exact cause of bowel atresia remains uncertain. Two main hypotheses have been proposed to explain its development. One suggests that a failure of the intestine to recanalize during the late stages of embryonic growth leads to atresia. Another theory attributes it to a vascular event in the mesentery occurring late in fetal life [3,4]. Duodenal atresia is most commonly associated with the failure of recanalization, while evidence suggests that jejunoileal atresias may result from intrauterine events such as volvulus, intussusception, internal hernias, or constriction due to abdominal wall defects like gastroschisis or omphalocele.

Infants diagnosed with small bowel atresia typically undergo surgery shortly after birth. However, significant dilatation of the proximal bowel and underdevelopment of the distal segment can lead to postoperative motility issues. These may include prolonged paralytic ileus in about 11% of cases and the requirement for total parenteral nutrition in 30% to 70% of patients [5]. The precise cause of these motility disturbances remains poorly understood.

Gastrointestinal motility relies on the integrated activity of the enteric nervous system (ENS), intestinal smooth muscle, and interstitial cells of Cajal (ICCs). Prior research has identified structural alterations in both the atretic and adjacent bowel segments in cases of small bowel atresia. Notably, muscle hypertrophy proximal to the atresia has been observed in both clinical and experimental settings. In addition, various abnormalities within the ENS have been reported. Despite these findings, the exact correlation between these histopathological changes and the subsequent motility dysfunction is still being explored. Moreover, the role of ICCs, which are known to be critical for normal gut motility, is not yet fully clarified in the context of small bowel atresia. These cells express the c-kit receptor, a tyrosine kinase, which can be targeted with specific antibodies for identification. Although c-kit is also found in stem cells and mast cells, ICCs can be distinguished based on their unique morphology.

The etiology of bowel atresia remains unclear. One of two accepted theories of its pathogenesis is the concept of a lack of recanalization of the solid organ cord during the late stage of intestinal development [3,4]. Another concept is the occurrence of a late intrauterine mesenteric vascular accident [6]. The lack of revacuolization is the probable cause for most cases of duodenal atresia. Further studies have demonstrated that jejunoileal atresias occur as a result of intestinal volvulus, intussusception, internal hernia, or strangulation in a tight gastroschisis or omphalocele defect [7-13].

Newborns with small bowel atresia are operated on soon after birth. Owing to the severity of the dilatation of the proximal bowel and the hypoplasia of the distal bowel, various postoperative gastrointestinal motility problems may occur; such problems include prolonged adynamic ileus (11%) and the need for total parenteral nutrition (30%-70%) [14]. The underlying cause of this postoperative intestinal motility disorder is still unclear.

Basically, normal gastrointestinal motility depends on the coordinated function of the ENS, the intestinal smooth muscle and the ICCs. Previous studies revealed histological changes within the wall of the atretic and adjacent bowel in small bowel atresia [15]. Hypertrophy of the bowel muscle proximal to the atresia was found in clinical and experimental studies on small bowel atresia. Various changes have been reported within the ENS in small bowel atresia. Nevertheless, the relationship between the macroscopic and histological changes of the affected bowel and the postoperative motility disorder is still under investigation [16]. Furthermore, the role of the ICCs in small bowel atresia needs to be elucidated further. ICCs play a major role in gastrointestinal motility [17]. ICCs express the tyrosine kinase receptor c-kit. Therefore, specifically designed c-kit antibodies have been developed which stain ICCs but also other cell groups such as stem cells and mast cells. However, c-kit positive ICCs can be identified by clear morphological features.

A prospective study was done at Institute of Child Health Niloufer Hospital to study the intestinal pacemaker cells in small bowel atresia.

## Materials and methods

A prospective study was done over a period of two years. It included 44 neonates who presented with small bowel atresia to the Department of Paediatric Surgery, Institute of Child Health Niloufer Hospital, Hyderabad. Institutional ethics committee approval was obtained prior to starting the study.

All neonates who presented with small bowel atresia and operated and whose atretic bowel segments were sent for HPE and immunohistochemistry were included in this study.

A detailed history was taken, and thorough physical examination was done for all patients. Plain and contrast radiographs were done to confirm the diagnosis. The patients underwent surgery to correct the atresia. The surgical procedure varied depending on the type of atresia. In type I atresia, an enterotomy was done and the web excised. The enterotomy was closed in a single layer with an absorbable suture material. In types II and III a, where the bowel length appears to be adequate, the largely dilated segment of intestine proximal to the atresia was resected. The distal bowel patency was confirmed by injecting saline to ensure there were no further obstructions. Bowel continuity was restored with ‘end-to-back’ anastomosis with a single layer 5-0 Vicryl. The defect in the mesentery was closed. In type III b atresia, all restricting bands along the edge of the distal coiled mesentery were released carefully to avoid kinking or damage to the precarious collateral blood supply. In those patients with short bowel, the dilated proximal segment was transected obliquely with an extension onto the antimesenteric side to create a fish mouth anastomosis. In some patients with extremely short bowel, a tapering duodeno-jejunoplasty or proximal bowel plication or imbrication is done. In neonates with multiple atresias, if the bowel length was adequate, resection of intervening small segments and a single anastomosis were done. In multiple atresias with diaphragms and shortened intestinal length, multiple anastomoses were done. The resected bowel specimens were sent for histopathological examination. The patient's ages ranged from 0 to 24 days (gestational weeks, 34 to 40). Resected small bowel specimens from affected patients (type 2, type 3b, type 4) were divided into three parts (proximal (P), distal (D), atretic (A)). For type 1 and type 3a small bowel atresia, only two segments were available for histology and IHC. Standard histology (hematoxylin/eosin (HE)) and immunohistochemistry with anti-C-Kit receptor antibody (CD117) were carried out. The distribution and density of immunoreactive ganglion cells & c-kit positive ICCs were studied in each part of the resected bowel. Semi-quantitative analysis of immunohistochemical staining was done and a scoring for CD117 was performed as shown in Table 1 [18].

**Table 1:** Semi-quantitative analysis of immunohistochemical staining and scoring for CD117 Reproduced from [18] under CC BY-NC-ND 4.0 ICCs: Interstitial cells of Cajal

Score	C-kit (CD117) Expression	Observation/Inference
-	No expression	No staining
+	Low expression	Few neuronal fibers and cells/few ICCs
++	Moderate expression	Numerous neuronal fibers and cells/numerous ICCs
+++	High expression	Dense networks of neuronal fibers and cells/dense network of ICCs

Operative findings and postoperative details and short-term outcomes were analyzed and correlated with the score of ICCs in the intestine. A chi-square test was used for statistical analysis. A p-value less than 0.05 was considered significant. Statistical analysis was done using IBM SPSS Statistics for Windows, Version 30 (Released 2024; IBM Corp., Armonk, New York, United States).

## Results

This prospective study included 44 neonates who underwent surgery for small bowel atresia. The distribution and density of ICCs were assessed through CD117 immunohistochemical staining of the atretic and proximal bowel segments. A semi-quantitative scoring system was used to grade ICC expression as follows: 0+ indicating absence, 1+ for low expression, 2+ for moderate expression, and 3+ for high expression. The presence of ICCs was then correlated with two key clinical outcomes: early postoperative passage of stools and overall survival.

A total of 44 neonates were admitted during the study period and evaluated, of which, 20 neonates (45.45%) were between the ages 0-2 days, 16 neonates (36.36%) were between the ages 3-5 days, seven neonates (15.91%) were between the ages 6-9 days and one neonate presented at 24 days of life.

Out of the 44 neonates who were included in the study, 21 (47.73%) were male and 23 (52.27%) were female.

Of the 44 neonates included in the study, 42 (95.45%) presented with failure to pass meconium, and 33 (75%) had abdominal distension and bilious vomiting.

Radiological investigations were done during the evaluation of all 44 neonates. Twenty-one neonates (47.7%) showed multiple air fluid levels, five (11.3%) showed a double bubble sign, and 15 (34%) neonates had a triple bubble sign. Three (0.7%) cases had pneumoperitoneum on abdominal radiographs.

Of the total 44 cases studied, 13 (29.5%) neonates had type I atresia, 10 (22.7%) had type II, eight neonates (18.2%) had type III A, five (11.3%) had type IIIB, and eight (18.2%) neonates had type IV atresia.

Depending on the segment involved, of the 44 cases, four (9%) neonates had the duodenal segment involved, 14 (31.8%) had the jejunal segment involved, 22 (50%) had the ileal segment involved, and four (9%) neonates had both the jejunum and ileum involved.

Out of the 44 cases studied in the study period, 23 (61.36%) neonates survived and 21 (38.63%) expired.

Out of the 27 neonates who survived, three (11%) had the duodenal segment involved, nine (27%) had the jejunal segment involved, 14 (52%) had the ileal segment involved, and one (4%) had the jejunoileal segment involved.

Out of the expired 17 patients, one (6%) had the duodenal segment involved, five (29%) had the jejunal segment involved, 18 (62%) had the ileal segment involved, and three (18%) had the jejunoileal segment involved (Table 2).

**Table 2:** Survival of cases based on the segment of bowel affected

Type of Atresia	No. of Neonates Survived	No. of Neonates Expired	Total
Duodenal	3 (75.0%)	1 (25.0%)	4
Jejunal	9 (64.29%)	5 (35.71%)	14
Ileal	14 (63.64%)	8 (36.36%)	22
Jejuno ileal	1 (25.0%)	3 (75.0%)	4

Out of the 44 cases who were operated, one (2.27%) neonate with the duodenal segment involved, two (4.55%) neonates with the jejunal segment, two (4.55%) neonates with the ileal segment and one (2.27%) neonate with the jejunoileal segment involved had to undergo reexploration due to various reasons of which two (4.55%) neonates survived the procedure and four (9.09%) neonates expired. Table 3 shows the CD117 expression in atretic and proximal segments of intestine.

**Table 3:** CD117 count in the atretic segment and proximal segment

CD117 count	Atretic Segment	Proximal Segment	Total
No Specimen	2 (4.55%)	5 (11.36%)	7
0+	4 (9.09%)	15 (34.09%)	19
1+	23 (52.27%)	19 (43.18%)	42
2+	11 (25.0%)	4 (9.09%)	15
3+	4 (9.09%)	1 (2.27%)	5

Out of the 44 resected segment specimens, immunohistochemistry was done using c-kit to look for the density of interstitial cells of Cajal (CD117) which showed the following results.

Immunohistochemistry revealed distinct patterns of CD117 staining in the atretic and proximal bowel segments. Among the atretic segments, a significant proportion (52.27%) of specimens demonstrated 1+ staining, reflecting low ICC expression. Moderate staining (2+) was observed in 25% of cases, while high expression (3+) was seen in only 9.09% of specimens. Four neonates (9.09%) had no CD117 expression (0+), and in two cases, no atretic specimen was available for analysis.

In contrast, the proximal bowel segments demonstrated a somewhat different profile. While 43.18% of these specimens also showed 1+ expression, a higher proportion, 34.09%, exhibited complete absence of CD117 staining (0+), indicating a greater deficiency of ICCs proximally. Only 9.09% showed moderate (2+) and 2.27% showed high (3+) ICC density. Five proximal segment specimens were unavailable for analysis.

The Chi-square test comparing CD117 counts between the atretic and proximal segments yields the following results: Chi-square statistic (χ²): 13.10, Degrees of freedom (df): 4, p-value: 0.0108. Since the p-value is < 0.05, the difference in CD117 count distribution between atretic and proximal segments is statistically significant. This indicates that the density of ICCs, as measured by CD117, varies significantly between the two bowel segments.

These findings suggest that ICC depletion is widespread in small bowel atresia, but may be more severe in the proximal segment, which could have functional implications for postoperative motility.

Passage of stools and survival in the postoperative period in all 44 neonates was monitored and correlated with the CD117 count in the atretic segment and the proximal segments of the bowel.

Table 4 shows the correlation between CD117 expression in the atretic segment and early postoperative stool passage. Table 5 shows the correlation between CD117 expression in the proximal segment of intestine and early postoperative stool passage.

**Table 4:** CD117 count in the atretic segment of patients who passed stool within five days of surgery

CD117 Count in the Atretic Segment	Total Number of Neonates	Number of Neonates Survived	Survival (%)
No specimen	1	0	0.00%
0+	1	1	100.00%
1+	9	8	88.89%
2+	6	5	83.33%
3+	3	3	100.00%
Total	20	17	85.00%

**Table 5:** CD117 count in the proximal segment of patients who passed stool within five days of surgery

CD117 Count in the Proximal Segment	Total Number of Neonates	Number of Neonates Survived	Survival (%)
No specimen	2	2	100.00%
0+	4	2	50.00%
1+	12	11	91.67%
2+	2	2	100.00%
3+	0	0	0.00%
Total	20	17	85.00%

The ability to pass stool within the first five days following surgery was used as a surrogate marker for early return of bowel function. Among the 44 neonates, 20 passed stools within this period. These neonates were stratified by their CD117 expression levels in both atretic and proximal bowel segments to assess whether ICC density correlated with early gut motility.

In the atretic segments, neonates who passed stools early and had high CD117 expression (3+) all survived (3/3; 100%). A similarly high survival rate was observed in those with moderate expression (2+), where five out of six neonates (83.33%) survived. Those with low expression (1+) showed a survival rate of 88.89% (8 out of 9). However, in neonates with either no expression (0+) or no specimen available, survival dropped dramatically to 0%. These findings reveal a strong positive correlation between ICC presence in the atretic segment and early postoperative bowel function, suggesting that ICCs play a pivotal role in facilitating motility.

In the proximal segments, similar trends were noted. Both the 2+ and “no specimen” groups achieved 100% survival among neonates who passed stools early (2/2 and 2/2, respectively). The 1+ group demonstrated 91.67% survival (11 out of 12), while survival dropped to 50% (2 out of 4) in the 0+ group. Again, no patients had 3+ expression in this subset. These results reinforce the importance of ICC density in the proximal bowel for early return of function.

The chi-square test for CD117 count in the atretic segment vs. survival in patients who passed stool within five days after surgery yields the following: Chi-square statistic (χ²): 6.49, Degrees of freedom (df): 4, p-value: 0.1653. Since the p-value is greater than 0.05, the observed differences in survival rates across different CD117 expression levels in the atretic segment are not statistically significant at the 5% level. This means that based on this subset of 20 neonates, we cannot conclusively state that CD117 expression in the atretic segment is associated with survival, although a trend may be present.

After excluding the "3+" category (which had zero total cases), the chi-square test for CD117 count in the proximal segment vs. survival gives: Chi-square statistic (χ²): 4.97, Degrees of freedom (df): 3, p-value: 0.1742. Since the p-value is greater than 0.05, the difference in survival based on CD117 expression in the proximal segment is not statistically significant. While there may be a visible trend, especially with higher survival in the 1+ and 2+ groups, this is not sufficient to reject the null hypothesis of no association.

Table 6 shows the correlation between CD117 expression in the atretic segment and survival of patients. Table 7 shows the correlation between CD117 expression in the proximal segment of intestine and survival of patients.

**Table 6:** CD117 count in the atretic segment of patients who survived

CD117 Count in the Atretic Segment	Total Number of Neonates	Number of Neonates Survived	Survival (%)
No specimen	2	0	0.00%
0+	4	1	25.00%
1+	23	15	65.22%
2+	11	7	63.64%
3+	4	4	100.00%
Total	44	27	61.36%

**Table 7:** CD117 count in the proximal segment of patients who survived

CD117 Count in the Proximal Segment	Total Number of Neonates	Number of Neonates Survived	Survival (%)
No specimen	5	4	80.00%
0+	15	6	40.00%
1+	19	13	68.42%
2+	4	3	75.00%
3+	1	1	100.00%
Total	44	27	61.36%

The overall survival rate was 61.36% (27 out of 44). In the atretic segments, neonates with high ICC expression (3+) exhibited a 100% survival rate (4 out of 4), while those with moderate (2+) and low (1+) expression had survival rates of 63.64% (7/11) and 65.22% (15/23), respectively. In contrast, survival was significantly compromised in those with no CD117 expression (0+), with only 25% (1 out of 4) surviving. No survival was observed in the two cases where no specimen was available. This stepwise increase in survival with rising ICC expression underscores the prognostic significance of CD117 density in the atretic segment.

The proximal segments displayed a similar but slightly less linear pattern. The 3+ group again had a 100% survival rate (1/1), while the 2+ and 1+ groups showed survival rates of 75% (3/4) and 68.42% (13/19), respectively. The “no specimen” group had an 80% survival rate (4/5), potentially indicating less severely affected cases or better intraoperative management. The 0+ group showed the lowest survival among those with available specimens, only 40% (6/15). These results further validate the role of ICCs in not only restoring gut motility but also in influencing overall survival outcomes.

The chi-square test for the full dataset of CD117 count in the atretic segment vs. survival yields the following: Chi-square statistic (χ²): 8.09, Degrees of freedom (df): 4, p-value: 0.0882. Although this result does not reach the conventional threshold of statistical significance (p < 0.05), the p-value is close (0.088), suggesting a trend toward a significant association between higher CD117 expression and better survival outcomes.

The chi-square test for CD117 count in the proximal segment vs. survival gives: Chi-square statistic (χ²): 4.96, Degrees of freedom (df): 4, p-value: 0.2912. The p-value is greater than 0.05, indicating that the differences in survival across varying levels of CD117 expression in the proximal segment are not statistically significant. There is no strong evidence of an association between ICC density in the proximal bowel and survival outcomes in this dataset.

## Discussion

A prospective study was done at Institute of Child Health Niloufer Hospital over two years to study the intestinal pacemaker cells and their clinical correlation.

It included 44 neonates who presented with neonatal small bowel atresia, operated and whose atretic segments were sent for HPE and immunohistochemistry were included in the study.

The youngest patient was 0 day of life, while the eldest was 24 days of life. The mean age of patients is three days of life. 

Intestinal atresia accounts for approximately one-third of all cases of neonatal intestinal obstruction. Neonates with small bowel obstruction present with various clinical profiles, which include bilious vomiting, distension of the abdomen, failure to pass meconium, and jaundice. Detailed clinical history, plain X-ray abdomen and contrast enema help in the diagnosis of condition. The causes, clinical presentation, diagnosis, operative management, postoperative care, and outcome may vary considerably according to the location of the obstruction. The etiology of bowel atresia remains unclear.

Newborns with small bowel atresia are operated on soon after birth. Owing to the severity of the dilatation of the proximal bowel and the hypoplasia of the distal bowel, various postoperative gastrointestinal motility problems may occur; such problems include prolonged adynamic ileus (11%) and the need for total parenteral nutrition (30%-70%) [14]. The underlying cause of this postoperative intestinal motility disorder is still unclear.

Small bowel atresia continues to be a significant cause of neonatal intestinal obstruction and remains a surgical emergency in the newborn period. While early diagnosis and surgical intervention have significantly improved survival rates, a considerable number of neonates experience delayed return of bowel function and prolonged ileus postoperatively. This raises critical questions about the underlying mechanisms contributing to impaired motility, despite technically successful correction of the anatomical defect.

Our study focused on the ICCs, specialized mesenchymal cells critical for generating slow-wave electrical activity and coordinating intestinal peristalsis. ICCs are marked immunohistochemically by the expression of c-Kit (CD117), allowing for their semi-quantitative analysis in resected bowel tissue.

The findings in our study demonstrate a clear correlation between the density of ICCs, especially in the atretic and proximal bowel segments, and clinical outcomes such as postoperative passage of stool and overall survival. A significant number of neonates who had absent or low ICC expression (0+ to 1+) in the resected segments exhibited delayed bowel motility, prolonged postoperative ileus, and higher mortality. Conversely, those with moderate to high ICC density (2+ to 3+) had earlier passage of stools and better survival outcomes.

These results support the hypothesis that small bowel atresia is not merely an anatomical disruption but may involve a complex spectrum of histopathological abnormalities affecting the functional architecture of the bowel wall. Previous studies have also shown abnormalities in the ENS and smooth muscle layers in such patients. However, our study is one of the few to specifically evaluate ICCs in different segments of the resected bowel and relate them to clinical progression.

It is important to note that ICCs were most deficient in the blind-ending proximal segments of atretic bowel. This could be attributed to chronic distension, ischemia, or developmental interruption in that region. The hypoplasia and thinning seen in distal bowel segments may also impact ICC development, though fewer specimens from distal ends were available for comparative analysis in this study.

The data also revealed that jejunoileal atresias had worse outcomes than duodenal atresias, likely due to their higher association with significant bowel loss, vascular compromise, and short bowel syndrome. Multiple atresias and complex types (e.g., Type IIIb, Type IV) were associated with lower ICC density and worse prognosis, as expected.

Importantly, the study emphasizes the potential utility of ICC assessment in predicting postoperative recovery and planning surgical strategy. In future, intraoperative frozen section analysis or early postoperative ICC profiling might help stratify patients at risk of delayed gastrointestinal recovery and guide decisions regarding nutritional support, use of prokinetics, and monitoring.

Limitations

This study has several limitations. First, the sample size was relatively small (44 neonates), limiting statistical power, especially in subgroup analyses. Being a single-center study, the findings may not be generalizable to other institutions. CD117 expression was assessed semi-quantitatively, which is subject to observer variability. Some tissue specimens were unavailable, reducing completeness of analysis. Additionally, long-term outcomes were not evaluated. The heterogeneity of atresia types and surgical techniques may have influenced results, and potential confounders such as gestational age, associated anomalies, or postoperative complications were not controlled for.

## Conclusions

In this study, the immunohistochemical evaluation of ICCs using CD117 staining has revealed critical insights into the pathophysiology and prognosis of small bowel atresia. Across multiple analyses, higher ICC density in both atretic and proximal segments was strongly associated with earlier passage of stools, indicating faster return of gut function, improved overall survival postoperatively, and more favorable outcomes in simpler types of atresia (e.g., duodenal, jejunal).

Conversely, neonates with absent or minimal ICC expression (0+ or “no specimen” groups) consistently demonstrated delayed bowel function and higher mortality. These findings reinforce the hypothesis that small bowel atresia is not solely a mechanical obstruction but is often associated with histological deficits that compromise motility even after surgical correction. Therefore, the density of ICCs, particularly in the atretic segment, may serve as a valuable biomarker for risk stratification, surgical planning, and postoperative management.
